# The emergence of the ectodysplasin pathway

**DOI:** 10.1515/biol-2025-1345

**Published:** 2026-07-14

**Authors:** Wilfred Donald Stein, Thomas Litman

**Affiliations:** Silberman Institute of Life Sciences, Hebrew University, Jerusalem, 91904, Israel; Department of Immunology and Microbiology, University of Copenhagen, 2200, Copenhagen N, Denmark

**Keywords:** EDA, EDAR, EDARADD, skin appendage placodes

## Abstract

The genes coding for the proteins of the ectodermal dysplasin pathway, *EDA,* EDAR and *EDA2R* are found in genomes of animals that long predate vertebrates. We show that *EDA, EDAR* and *EDA2R* are expressed in the sea anemone. A fourth gene in the pathway, *EDARADD*, only appears with the cyclostomes. With the appearance of *EDARADD,* the ectodermal dysplasin system was now complete, and the skin appendages emerged, first in the form of the cyclostome’s keratinized proto-teeth, until finally, feathers and then mammalian hair and eccrine glands evolved. We elaborate on the specific roles of the ectodermal dysplasin pathway proteins: EDA releases its extracellular circulating component, switching on the pathway at the appropriate stage in embryological development. EDA binds to EDAR, whose location in the embryo has been specified by a reaction-diffusion system. The EDA/EDAR complex now binds to intracellular EDARADD, directing the expression of downstream proteins that orchestrate the mechanics of the cell movements, building the placodes and the appropriate skin appendages.

## 1 Introduction

The embryological development of teeth, scales, feathers, hair, sweat and mammary glands, the epidermal appendages, begins with the formation in the epidermis of a placode (from the Greek plak- something flat), a thickening of the epidermis. The subsequent invagination of this thickened layer forms a crevice, out of which the epidermal appendages will emerge. The development of a skin appendage placode is controlled by three genes of the ectodysplasin pathway: *EDA, EDAR* and *EDARADD* [1] (The term “placode” is also used to describe the embryological entities which lead to the development of the sensory organs that monitor the senses of smell, sight and hearing [2]. We will not discuss these sensory placodes).

The gene *EDA, Ectodysplasin A*, codes for the transmembrane protein EDA that can be cleaved by the protease furin to produce a secreted form. This secreted form binds to its receptor EDAR, the Ectodysplasin A receptor, a membrane-bound protein, which in turn activates cytoplasmic EDARADD, the EDAR-associated Death Domain protein. Diagrams of structural features of EDA and EDAR are shown in Supplementary Figures S1 and S2, respectively. These features include the transmembrane domain, the glycosylation sites and the disulfide bonds and, for EDA, the furin-cleavage site. EDARADD, after binding to TRAF6, activates the downstream NEMO/IKK signal. This latter induces the NFκB system genes that control the cellular movements which build the placode. Mutations in either *EDA*,* EDAR,* or *EDARADD* give rise to the clinical syndrome of ectodermal dysplasia, a genetic disorder characterized by defective development of hair, teeth, sweat glands and the meibomian glands (tear-producing glands) of the eye. The *EDA* gene is the most common ectodermal dysplasia-causing gene – 58 % of cases in a study by Cluzeau et al. [3] (Fingernails are often listed as being an additional appendage whose presence or absence is controlled by the genes of the ectodermal dysplasin pathway, but this is not entirely correct. Maier-Wohlfart et al. [4], studying a cohort of children with ectodermal dysplasia, found that none of the 161 children with mutant EDA had nail defects, although some of the rarer cases having defective EDAR or EDARADD genes did show nail abnormalities). Depending on the nature of the mutation in the ectodermal dysplasin pathway genes, the clinical picture can range from the complete absence of hair, teeth and sweat glands to the appearance of a small number of, or deformed teeth (For an update on the clinical findings and classification of the ectodermal dysplasias see Peschel et al. [5]). Mutations in these genes can be found in other vertebrates with resulting deformations of appropriate epidermal appendages, such as scales in fish, and snakes and feathers in chickens.

Some clues as to what these genes may be doing in functions other than that of controlling the development of placodes, come from the following considerations:

People who suffer from ectodermal dysplasia can have defects also in certain skeletal properties, such as a characteristic reduced facial length, a depressed nasal bridge and growth alterations in the cranial base [6], 7]. A mutation in the EDA gene is associated with defects in the tailbones of mice carrying that mutation [8]. Thus, a connection seems to exist between bone metabolism and the genes of the ectodysplasin pathway. Interestingly, EDA and EDAR seem to control the development of the rakers of the gill arches in the stickleback fish [9], these being a segmentally reiterated set of dermal bones. In the Mandarin fish, He et al. [10] demonstrate a very clear absence of these rakers in the fish doubly mutant for EDAR. Now the gill arches are the evolutionary forerunners of the lower jaws of the higher vertebrates [11]. These findings concerning the gill rakers can perhaps help lead to an understanding of the role of the ectodermal dysplasin genes in the bone formation symptoms of ectodermal dysplasia patients.

In quite a different context, an important recent finding is that EDA was identified as a hepatokine, a liver-secreted protein, that is increased in the liver and plasma of obese mice and, in humans, causes skeletal muscle insulin resistance [12]. This is again a finding that shows that EDA can have a function that is quite independent of the ectodysplasin pathway.

In what follows we will record the evidence that the *EDA, EDAR* and *EDA2R* genes are present in animals that long predate the cyclostomes (the lampreys and hagfishes). Finally, we discuss new evidence that points to the specific role of EDA and EDAR in positioning the placodes, leaving the EDARADD gene to be the functional determinant of the mechanism of placode formation.

## 2 Methods and definitions

Searches in other animal species for homologs of the proteins of the ectodermal dysplasin pathway of *H. sapiens,* as well as searches for homologs of other proteins, were performed using the protein BLAST (Basic Local Alignment Search Tool) program of the NCBI: https://blast.ncbi.nlm.nih.gov/Blast.cgi with the program’s default parameters: Max target sequences 100, Expect threshold 0.05, Word size 5, Max matches in a query range 0, Matrix BLOSUM62, Gap Costs Existence: 11 Extension: 1, conditional compositional score matrix adjustment, No Low complexity regions filter. Proteins with Expect values lower than, or equal to 10^−3^ compared with the bait in a 2-sequence comparison were defined as **structurally similar** to the bait used in the BLAST search. Alignments between protein sequences and a resultant illustrative phylogram were established using the COBALT (Constraint-Based Alignment Tool) aligner at the NCBI: https://www.ncbi.nlm.nih.gov/tools/cobalt/cobalt.cgi?CMD=Web.

Data on the expression of genes in sea anemones were extracted from the INvERTx Embryo-Regen plotter (ircan.org) database.

Human gene expression data for 50 tissue types were downloaded from The Human Protein Atlas project version 24.0 (https://www.proteinatlas.org/).

## 3 Results

### 3.1 Appearance of the genes of the ectodermal dysplasin pathway in animals from the echinoderms to the bony fish

We performed BLAST searches, using the *H. sapiens* EDA, EDAR, EDA2R and EDARADD proteins, looking for antecedents of these proteins in animals that appeared earlier in evolution than the cyclostomata.

The sequences of all the proteins discussed in the following section are depicted in Supplementary Figure S3. Included in the figure are alignments between the sequences of each human protein specified and the corresponding protein in an earlier-appearing animal.

In the case of EDA, we found its presence in the genome of a tunicate as the ectodysplasin-A (*Phallusia mammillata*) with gene annotation CAB3240801.1 and a 2-sequence Expect value of 2^−18^ against the EDA of *H. sapiens*.

EDAR was found in the genome of the Branchiostomatidae as the EDAR of the lancelet *Branchiostoma lanceolatum* with gene annotation CAH1267392.1 and a 2-sequence Expect value of 2^−15^ against the EDAR of *H. sapiens*.

EDA2R was found in the Echinodermata as the tumor necrosis factor receptor superfamily member 27 isoform X2 of *Strongylocentrotus purpuratus* with annotation XP_030833294.1 and a 2-sequence Expect value of 7^−19^ against the EDA2R of *H. sapiens*.

A homolog of EDARADD was not found in the Tunicata, the sister clade to the vertebrata. Nor was one found in the earlier-appearing Branchiostomatidae.

The gene TNFRSF19 is a TNF receptor, homologous with EDAR in its ligand-binding domain, and is expressed in an overlapping pattern [13]. It seems to act redundantly with EDAR. The double mutant of the murine TNFRSF19 in a wild type/mutant cross showed defects of hair production in the crowns of the mice. A search for TNFRSF19 in the genomes of the cyclostomes failed to find a homolog of TNFRSF19, but one was found in the later-appearing cartilaginous fish.

Interestingly Steichele and her colleagues [14] found that homologs of EDA and EDAR (HyTNF and HyTNFR, respectively) were expressed in Hydra, and also in the sea anemone [15].

The EDARADD gene of *H. sapiens* is found in three different isoforms. Sadier et al. [16] found that one form appeared only with the mammals, and was then lost in certain lineages, suggesting that it plays a specific role in the diversification of the function of the EDA pathway.

It is the Death Domain that is involved in the binding between EDAR and its cytoplasmic partner EDARADD. We searched in BLAST with the death domain sequence:

>death domain

DVIRIKLDPCHPTVKNWRNFASKWGMSYDELCFLEQRPQSPTLEFLLRNSQRTVGQLMELCRLYHRADVEKVLRRWVDEE

The top hit in the Tunicata was the myeloid differentiation primary response protein MyD88 of the white warty sea squirt *P*. *mammillata*, having accession number CAB3264104.1 and an Expect value of 8e-08 in a BLAST 2-sequence comparison with that death domain sequence.

### 3.2 Expression of the genes of the ectodermal dysplasin pathway

It is to be expected that one would find the genes of the Ectodermal Dysplasin pathway expressed in those organs where placodes are to be found, i.e. in the tissues from which the teeth, hair, and the eccrine glands arise. We wondered, however, whether some or all of these genes might be found in other tissues of the body. A summary of the results of our search, querying The Human Protein Atlas, is given in Table 1 below.

**Table 1:** Average gene expression and rank (out of 20,162 human genes) across 50 human tissues for EDA, EDAR, EDA2R, and EDARADD. For the full details on which this summary Table is based see the human gene expression data for 50 tissue types as downloaded from the Human Protein Atlas project version 24.0 as referenced in the Supplementary Table S1.

Gene	Average expression	Rank	% Rank
EDA	2.566	14,671	27.2
EDAR	0.49	17,651	12.5
EDA2R	2.366	14,883	26.2
EDARADD	1.594	15,794	21.7

Overall, we see measurable but low expression of these genes; EDA and EDA2R are in the lower 26–27 % rank (out of the 20,162 human genes), EDARADD in the lower 22 % rank, and EDAR in the lowest 12.5 % rank.

Querying GeneCards: https://genealacart.genecards.org/Query returned a picture of low expression across all tissues, with no clear difference between the tissues.

In the previous section, we showed that EDA, EDAR, and EDA2R could be found as annotated sequences in the genome of animals that evolved before placodes appeared. We attempted to find out if one or all of these genes might be present as expressed genes in these animals. We chose to enquire at the sea anemone database which has a comprehensive, searchable tabulation of gene expressions in a number of different research study protocols.

As noted in the Methods section, access to expression of genes in sea anemones is available through the INvERTx Embryo-Regen plotter (ircan.org) database. In one series that we queried, regeneration of the mouth and tentacles was followed as a function of time (For a description of oral regeneration in the sea anemone *Nematostella vectensis* see Amiel et al. [15]).

To search this database for the gene EDA, we first BLASTed the genome of *N*. *vectensis* with the EDA gene from *H*. *sapiens*. The top hit with length comparable to that of human EDA was the gene ectodysplasin-A isoform X3 (*N*. *vectensis)* listed as sequence XP_048588342.1. A 2-sequence comparison of this gene with human EDA returned an Expect value of 9E-24.

Entering the sequence XP_048588342.1 into the sea anemone database returned a number of sea anemone genes with acceptable Expect values in comparison with that sequence.

**Figure 1:**
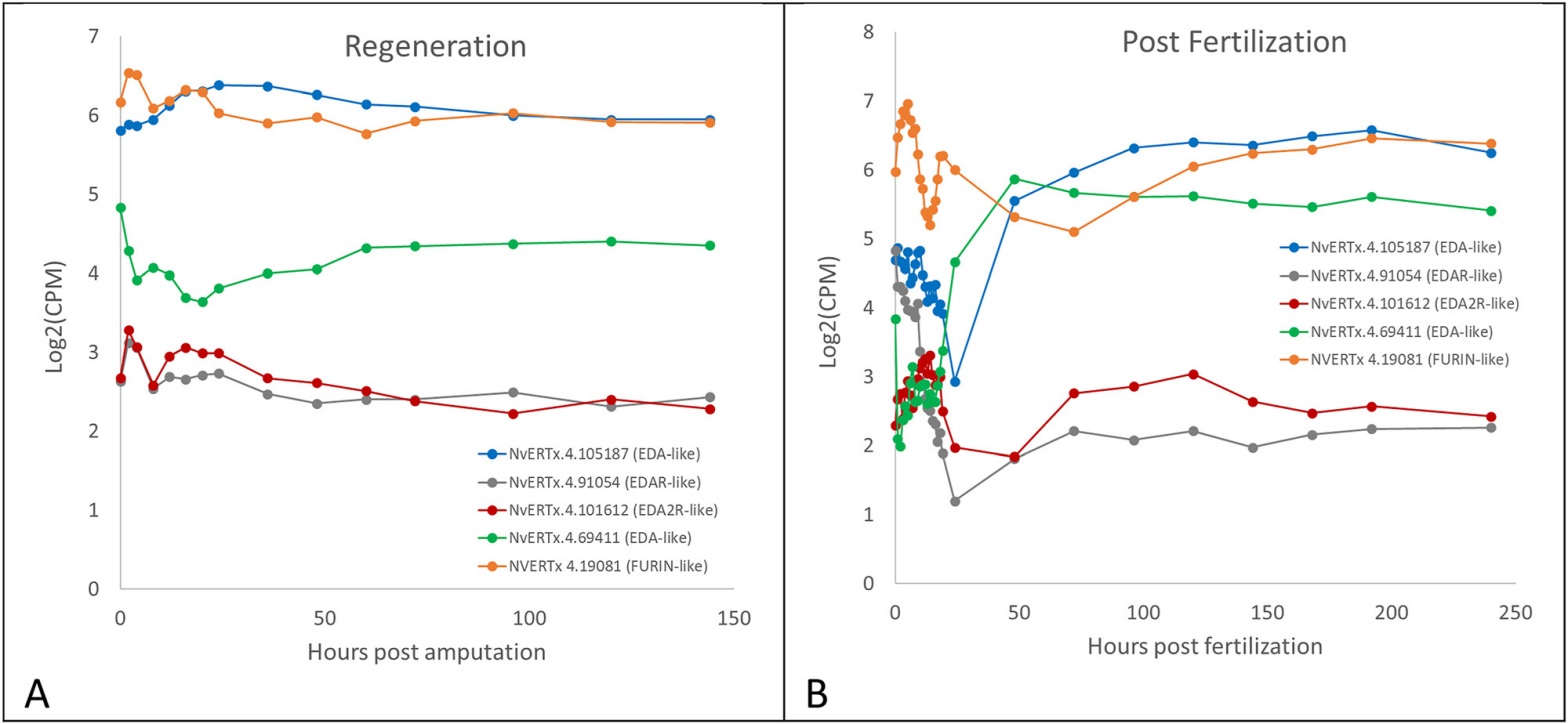
A. Regenerative and B. embryonic expression in sea anemone of NvERTx.4.105187 (overlapping with NvERTx.4.105188), NvERTx.4.91054, NvERTx.4.101612, NvERTx.4.69411, and NvERTx.4.19081 (data – see text – from https://nvertx.ircan.org/).

The two top hits, NvERTx.4.105187 and NvERTx.4.105188, with Expect values of 0.0 and 6E-118 respectively, correspond to the same gene (transcript), as they have the same expression values (that for NvERTx.4.105187 depicted in Figure 1 A and B) (The figure includes expression data for the gene NvERTx.4.69411 discussed below).

The expression of two other sea anemone genes, NvERTx.4.91054 (EDAR-like), and NvERTx.4.101612 (EDA2R-like) is also illustrated in Figure 1, and appears to be coordinated, both with respect to regeneration (Figure 1A) and post fertilization (Figure 1B). Additionally, the sea anemone homolog of furin (NvERTx.4.19081) is shown on the figure.

We again queried the NvERTx Embryo-Regen plotter (ircan.org) database, this time searching for ”ectodysplasin”. We received a single hit: **NvERTx.4.69411,** listed there with synonym XP_001638170. The expression of NvERTx.4.69411 was shown on Figure 1 above. BLASTing this against the genome of *N*. *vectensis* gave for the topmost annotated hit “ectodysplasin A *N*. *vectensis*”, listed as XP_048588379.1. A 2-sequence comparison of this gene with human EDA returned an Expect value of 4E-24.

Thus, we found evidence for the expression of *EDA, EDAR, EDA2R,* and *FURIN* in the sea anemone, both during its regeneration and in its development after fertilization.

## 4 Discussion

### 4.1 Roles of the genes of the ectodermal dysplasin pathway

It would appear that it is the EDAR gene that determines the spatial location of the products of the EDA pathway. Teeth, hair, sweat glands and also the scales and the rakers of the gill arches in fish are located in precise locations in the body of vertebrates, generally in an ordered pattern. Consider, for example, the whorl of pharyngeal teeth in the lamprey and hagfish or the teeth in the human, and the distribution of hair and sweat glands in our bodies. In an enlightening recent paper, Glover et al. [17] studied the mechanism that led to the pattern of fingerprints in humans and other animals. They showed first that fingerprints, with their very precise pattern, individual to each person, are also a product of the ectodermal dysplasin system. Indeed, one of the lesser-known symptoms of ectodermal dysplasia is the poor appearance of fingerprints, Verbov [18] suggests that this disturbance in the pattern of fingerprints could lead to improved diagnosis of ectodermal dysplasia and to the identification of carriers of mutations in the EDA pathway genes. Fingerprint ridges arise in development from epithelial buds which are analogous to the placodes that form hair and teeth. Primary fingerprint ridges express EDAR but differ from hair placodes in that they express lower levels of the WNT gene and higher levels of the BMP gene. Both genes are members of the cascade of genes that lead to the formation of hair and teeth. On the basis of gene expression findings on fingerprints, Glover and his colleagues (*loc. cit.*) modelled a Turing reaction-diffusion system involving EDAR, with WNT being an activator of epidermal ridge formation and BMP being an inhibitor. Their model was able to account in broad fashion for the patterns of fingerprints. On the basis of this study and the earlier embryonic patterning modellings of EDAR in mouse skin explants [19] and mouse teeth [20], one can suggest that it is EDAR that determines the location of placodes, the gene being expressed in response to pattern formation signals [21] formed by reciprocal inhibitory interactions between EDAR and BMP producing a Turing reaction-diffusion system. Others have explicitly followed this approach in analysing the pattern of scale distribution in the zebrafish [22] and scale patterning in corn snakes [23].

To understand the role of the EDA itself one must remember that this gene is a ligand member of the tumor necrosis superfamily, indeed being the gene *TNSF29*. The *TNSF* genes are membrane-bound and produce cytokines upon enzymatic release of the soluble portion of the gene. EDA itself is a hepatokine, being expressed in, and its circulating form being released from, the liver. Soluble EDA can be found circulating in the blood. A study by Bayliss et al. [12] showed that high levels of plasma EDA were associated with a worsening of non-alcoholic fatty liver in clinical cases. Awazawa et al. [24] showed that liver-derived EDA regulates systemic glucose metabolism, with EDA, acting as an hepatokine, contributing to impaired skeletal muscle insulin sensitivity in obesity. Thus, EDA is involved not only in the formation of the skin appendage placodes.

It is important to note that the defect resulting from a mutant EDA gene (this being the most common cause of ectodermal dysplasia) can be repaired by the intravenous injection of recombinant EDA into dogs [25] and, more recently by its ultrasound-guided intra-amniotic application to embryos in dogs [26], and also in humans [27]. Thus, EDA is a circulating signaling molecule that can access the developing placodes. EDA is then the trigger that enables the placode to complete its developmental program.

With EDA and EDAR present at defined epidermal locations in the body, these are in a position to recruit EDARADD, which on binding to EDAR is activated and itself can now bind the downstream genes of the ectodermal dysplasin pathway which provide the cellular mechanics for the building of the placodes. The sophisticated studies of Mikkola and her colleagues [28], 29] describe for us the morphological details of the progress by which the skin appendage placodes form hair and teeth. They show that cell compaction and centripetal migration are the main cellular mechanisms associated with hair placode morphogenesis. The cell migration is driven by actin dynamics: inhibition of actin remodeling suppressed placode formation. Importantly, their studies linked the cellular movements in placode formation directly with the downstream signaling molecules of the ectodysplasin pathway, NFκB and Wnt/β-cat.

### 4.2 The emergence of the ectodysplasin pathway

The earliest animals in evolution to show the presence of functional products of the Ectodermal Dysplasin pathway are the cyclostomes such as the hagfish and the lamprey which lack scales, jaws, and bony teeth, but do possess keratin-based teeth in their pharynx. In accord with this, the genomes of these animals contain all three genes of the ectodysplasin pathway. But this is not the case for animals lower in the evolutionary progression than the cyclostomes. Thus, for example, the genome of the tunicates contains EDA and also EDAR but not EDARADD. Clearly, the genes EDA and EDAR must be performing some function other than being involved in the formation of the placodes that form hair, skin and teeth. Indeed, the two genes EDA and EDAR have a very long evolutionary history. We show in the RESULTS section that EDA is present in the genome of the sea anemone, a member of the Cnidaria phylum, while both EDAR and EDA2R are also expressed in the sea anemone. The EDA of the sea anemone is likely to be membrane-bound since its sequence matches well the membrane-spanning portion of the human EDA. Accordingly, also present in the genome of, and expressed in, this animal is the gene for FURIN, a protease – one of whose many tasks in an organism is to release the secreted form of EDA in the chordates [30], 31]. Placodes, in the sense of thickenings of the epithelium, have been described in the development of the tentacles of the sea anemone, the morphogenetic movements here being associated with the genes *Notch* and *Delta* rather than with the genes of the ectodermal dysplasin pathway [32]. We found a homolog of *EDAR* (with a significant Expect value) to be present in the genome of the lancelet *B. lanceolatum*, suggesting that these genes might well have a role in this pre-vertebrate animal.

Thus, for the full ectodysplasin pathway to emerge in the lamprey it was only the *EDARADD* gene that still had to appear.

## 5 Conclusions

The skin appendage systems, teeth, hair and the eccrine glands, emerge in evolution as vertebrate inventions. Their embryological development is controlled by the proteins of the ectodermal dysplasin pathway, EDA, EDAR, EDA2R, and EDARADD. We show that *EDA* and the *EDAR* genes are found in the genomes of animals that long predate the vertebrates. Indeed, they are expressed in an animal as early as the sea anemone. It is plausible to assume that these proteins are performing some, at present unknown, function in these organisms. Indeed, both EDA and EDAR have actions in the human body other than in the embryological development of the skin appendages. The fourth gene in the pathway, EDARADD, only appears with the cyclostomes, at the dawn of the vertebrate clade. With the appearance of *EDARADD,* the ectodermal dysplasin system was now complete, and the skin appendages could emerge – first in the form of the keratinized proto-teeth of the cyclostomes, then as the calcified teeth of the conodonts and the later non-jawed and then jawed fish, and finally as the hair and eccrine glands of the mammals. We suggest specific roles of the proteins of the ectodermal dysplasin pathway: EDA releases its extracellular circulating component – which acting as a timing device, switches on the ectodermal dysplasin pathway at the appropriate stage in embryological development. EDA binds to EDAR, whose location in the embryo is then specified by a reaction-diffusion signalling system. The EDA/EDAR complex can now bind to intracellular EDARADD which directs the expression of the downstream proteins that bring about the mechanics of the cell movements, building the placodes and, eventually, the appropriate skin appendages.

## Supplementary Material

Supplementary Material

Supplementary Material

## References

[1] Biggs LC, Mikkola ML (2014). Early inductive events in ectodermal appendage morphogenesis. Semin Cell Dev Biol [Internet].

[2] Bassham S, Postlethwait JH (2005). The evolutionary history of placodes: a molecular genetic investigation of the larvacean urochordate *Oikopleura dioica*. Development.

[3] Cluzeau C, Hadj-Rabia S, Jambou M, Mansour S, Guigue P, Masmoudi S (2011). Only four genes [EDA1, EDAR, EDARADD, and WNT10A] account for 90% of hypohidrotic/anhidrotic ectodermal dysplasia cases. Hum Mutat.

[4] Maier-Wohlfart S, Aicher C, Willershausen I, Peschel N, Meißner U, Gölz L (2022). Congenital nail disorders among children with suspected ectodermal dysplasias. Genes [Basel].

[5] Peschel N, Wright JT, Koster MI, Clarke AJ, Tadini G, Fete M (2022). Molecular pathway-based classification of ectodermal dysplasias: first five-yearly update. Genes [Basel].

[6] Clauss F, Manière MC, Obry F, Waltmann E, Hadj-Rabia S, Bodemer C (2008). Dento-craniofacial phenotypes and underlying molecular mechanisms hypohidrotic ectodermal dysplasia [HED]: a review. J Dent Res.

[7] Goodwin AF, Larson JR, Jones KB, Liberton DK, Landan M, Wang Z (2014). Craniofacial morphometric analysis of individuals with x-linked hypohidrotic ectodermal dysplasia. Mol Genet Genom Med.

[8] Hill NL, Laib A, Duncan MK (2002). Mutation of the ectodysplasin-A gene results in bone defects in mice. J Comp Pathology [Internet].

[9] Glazer AM, Cleves PA, Erickson PA, Lam AY, Miller CT (2014). Parallel developmental genetic features underlie stickleback gill raker evolution. Evodevo.

[10] He S, Li L, Lv LY, Cai WJ, Dou YQ, Li J (2020). Mandarin fish [Sinipercidae] genomes provide insights into innate predatory feeding. Commun Biol [Internet].

[11] Fraser GJ, Hulsey CD, Bloomquist RF, Uyesugi K, Manley NR, Streelman JT (2009). An ancient gene network is co-opted for teeth on old and new jaws. PLoS Biol.

[12] Bayliss J, Ooi GJ, De Nardo W, Shah YJH, Montgomery MK, McLean C (2021). Ectodysplasin A is increased in non-alcoholic fatty liver disease, but is not associated with type 2 diabetes. Front Endocrinol [Lausanne].

[13] Pispa J, Pummila M, Barker PA, Thesleff I, Mikkola ML (2008). Edar and Troy signalling pathways act redundantly to regulate initiation of hair follicle development. Hum Mol Genet.

[14] Steichele M, Sauermann LS, König AC, Hauck S, Böttger A (2021). Ancestral role of TNF-R pathway in cell differentiation in the basal metazoan *Hydra*. J Cell Sci.

[15] Amiel AR, Johnston HT, Nedoncelle K, Warner JF, Ferreira S, Röttinger E (2015). Characterization of morphological and cellular events underlying oral regeneration in the sea anemone, *Nematostella vectensis*. Int J Mol Sci.

[16] Sadier A, Lambert E, Chevret P, Décimo D, Sémon M, Tohmé M (2015). Tinkering signaling pathways by gain and loss of protein isoforms: the case of the EDA pathway regulator EDARADD. BMC Evol Biol.

[17] Glover JD, Sudderick ZR, Shih BBJ, Batho-Samblas C, Charlton L, Krause AL (2023). The developmental basis of fingerprint pattern formation and variation. Cell.

[18] Verbov J (1970). Hypohidrotic [or anhidrotic] ectodermal dysplasia – an appraisal of diagnostic methods. Br J Dermatol.

[19] Mou C, Jackson B, Schneider P, Overbeek PA, Headon DJ (2006). Generation of the primary hair follicle pattern. Proc Natl Acad Sci USA..

[20] Sadier A, Twarogowskaid M, Steklikova K, Haydenid L, Lambert A, Schneiderid P (2019). Modeling edar expression reveals the hidden dynamics of tooth signaling center patterning. PLoS Biol.

[21] Wolpert L (2016). Positional information and pattern formation. Curr Top Dev Biol.

[22] Aman AJ, Fulbright AN, Parichy DM (2018). Wnt/β -catenin regulates an ancient signaling network during zebrafish scale development. eLife.

[23] Tzika AC, Ullate-Agote A, Zakany S, Kummrow M, Milinkovitch MC (2023). Somitic positional information guides self-organized patterning of snake scales. Sci Adv.

[24] Awazawa M, Gabel P, Tsaousidou E, Nolte H, Krüger M, Schmitz J (2017). A microRNA screen reveals that elevated hepatic ectodysplasin A expression contributes to obesity-induced insulin resistance in skeletal muscle. Nat Med.

[25] Casal ML, Lewis JR, Mauldin EA, Tardivel A, Ingold K, Favre M (2007). Significant correction of disease after postnatal administration of recombinant ectodysplasin A in canine X-linked ectodermal dysplasia. Am J Hum Genet.

[26] Margolis CA, Schneider P, Huttner K, Kirby N, Houser TP, Wildman L (2019). Prenatal treatment of X-linked hypohidrotic ectodermal dysplasia using recombinant ectodysplasin in a canine model. J Pharmacol Exp Ther.

[27] Schneider H, Schweikl C, Faschingbauer F, Hadj-Rabia S, Schneider P (2023). A causal treatment for X-linked hypohidrotic ectodermal dysplasia: long-term results of short-term perinatal ectodysplasin A1 replacement. Int J Mol Sci.

[28] Ahtiainen L, Lefebvre S, Lindfors PH, Renvoisé E, Shirokova V, Vartiainen MK (2014). Directional cell migration, but not proliferation, drives hair placode morphogenesis. Dev Cell.

[29] Ahtiainen L, Uski I, Thesleff I, Mikkola ML (2016). Early epithelial signaling center governs tooth budding morphogenesis. J Cell Biol.

[30] Elomaa O, Pulkkinen K, Hannelius U, Mikkola M, Saarialho-Kere U, Kere J (2001). Ectodysplasin is released by proteolytic shedding and binds to the EDAR protein. Hum Mol Genet.

[31] Roebroek AJM, Taylor NA, Louagie E, Pauli I, Smeijers L, Snellinx A (2004). Limited redundancy of the proprotein convertase furin in mouse liver. J Biol Chem [Internet].

[32] Fritz AE, Ikmi A, Seidel C, Paulson A, Gibson MC (2013). Mechanisms of tentacle morphogenesis in the sea anemone *Nematostella vectensis*. Development.

