# Corrigendum to “The emergence of the ectodysplasin pathway”

**DOI:** 10.1515/biol-2025-1395

**Published:** 2026-10-28

**Authors:** Wilfred Donald Stein, Thomas Litman

**Affiliations:** Silberman Institute of Life Sciences, Hebrew University, Jerusalem, 91904, Israel; Department of Immunology and Microbiology, University of Copenhagen, 2200 Copenhagen N, Denmark

**Original article**

Stein WD, Litman T. The emergence of the ectodysplasin pathway. Open Life Sciences. 2026;21(1):20251345. https://doi.org/10.1515/biol-2025-1345.

We correct our statements concerning the distribution and evolutionary origin of EDARADD and qualify the proposed relationship between its emergence and cyclostome toothlet development. Our original analysis excluded unannotated protein hits from further consideration. This selection criterion was too restrictive for identifying evolutionarily distant homologs. An earlier genomic survey reported EDARADD homologs in amphioxus within its immune-gene repertoire [1]. This finding appears in the article’s full text but is not retrieved by a PubMed search combining ‘EDARADD’ and ‘amphioxus’. These findings suggest that EDARADD-related proteins existed before cyclostomes evolved. The emergence of cyclostome keratinous toothlets may therefore reflect the recruitment of an existing signaling system for a new developmental function, rather than the first appearance of EDARADD.

In their genomic survey of the immune-gene repertoire of *Branchiostoma floridae*, Huang et al. [1] identified EDARADD homologs alongside FADD, CRADD, TRAF-family adaptors, and numerous TNF ligands and receptors. They discussed these proteins as potential components of the amphioxus TNF-signaling network within its extensively diversified innate immune system. Thus, EDARADD-related proteins had already been recognized in amphioxus in an immune-signaling context. This was a comparative genomic assignment, however, rather than an experimental demonstration of the immune function of an individual amphioxus EDARADD homolog [1].

Our subsequent sequence analyses identify EDARADD-related proteins in *B. belcheri* (XP_019641213.1) and *B. floridae* (XP_035677584.1). These records carry the automated descriptions “myosin tail region-interacting protein MTI1-like” and “uncharacterized protein,” respectively. Both predicted proteins contain 558 amino acids and show significant similarity to human EDARADD, concentrated in their C-terminal death-domain regions. Reciprocal comparisons support their assignment as EDARADD-related homologs, although their precise orthology and functional equivalence to vertebrate EDARADD remain unresolved. Such candidates should have been evaluated using sequence similarity and domain architecture irrespective of their annotation labels.

The immune context is relevant to the evolutionary interpretation. Several components associated with vertebrate EDARADD signaling also participate in older immune-signaling systems. In *B. belcheri*, TAK1 has been characterized in an innate-immune context: its expression responds to lipopolysaccharide stimulation, and its association with TAB1 has been demonstrated. These findings support the existence of functional downstream signaling machinery in amphioxus, although they do not establish its connection to the EDARADD-related protein [2].

Beyond the death-domain similarity, the N-terminal regions provide an additional indication of possible functional conservation. Experimental studies place human EDARADD’s interaction with TRAF6 in its N-terminal region. Structural studies of TRAF6 recognition identify the consensus **P-x-E-x-x-Z**, where x denotes any amino acid and Z is an acidic or aromatic residue. Inspection of the human, lamprey and lancelet sequences identifies the following matching motifs [3], 4].

**Table:** 

Species	Protein accession	Candidate TRAF6-binding sequence	Residues
*Homo sapiens*	Q8WWZ3	PVEDTD	27–32
*Petromyzon marinus*	XP_032830143.1	PVESDY	36–41
*Branchiostoma belcheri*	XP_019641213.1	PVESVD	181–186
*Branchiostoma floridae*	XP_035677584.1	PVESED	180–185

Residue positions refer to the specified protein sequences, numbered from the initiating methionine.

All four sequences retain the PVE core and satisfy the six-residue recognition pattern. They occur upstream of the death domain; the corresponding segments in the human and lancelet proteins fall within predicted intrinsically disordered regions. Such regions can accommodate short interaction motifs despite considerable variation in the surrounding sequence. These observations are consistent with conservation of a potential TRAF6-recruiting feature in EDARADD-related proteins.

Nevertheless, short motifs can occur by chance, and their presence does not itself demonstrate binding or equivalent molecular function. Direct interaction with TRAF6 has not been demonstrated for the lamprey and lancelet EDARADD-related proteins examined here. We therefore describe these sequences as **shared putative TRAF6-binding motifs**, rather than experimentally established conservation of a binding domain.

Our sequence searches and reciprocal comparisons also identify TRAF6 and TAK1/MAP3K7 homologs in both lamprey and lancelet, together with TAB2 in *P. marinus* and a TAB2/3-family protein in *B. belcheri*. The lancelet assignment should remain at the family level, because alternative protein models from one locus do not establish separate TAB2 and TAB3 genes. The demonstrated amphioxus TAK1–TAB1 association should likewise not be taken as evidence of TAK1–TAB2 binding.

In lamprey, a June 2026 preprint reports that TAK1 from *Lethenteron reissneri* associates with both TAB1 and TAB2 in transfected HEK293T cells. This provides additional evidence that downstream components can assemble into complexes but does not establish their recruitment by EDARADD or their involvement in toothlet development [5].

In the experimentally characterized mammalian pathway, EDARADD connects EDAR signaling to machinery involving TRAF6, TAB2 and TAK1, leading to IKK/NF-κB activation. The candidate TRAF6-binding motif should not be interpreted as a demonstrated binding site for all three downstream proteins. Furthermore, the canonical appendage-associated branch is EDA-A1–EDAR–EDARADD. EDA2R/XEDAR participates in a separate EDA-A2-responsive branch and is not an additional obligatory link in that sequence [6], [7], [8].

Taken together, the amphioxus immune-gene survey, the EDARADD-related sequences, their compatible TRAF6-binding motifs and the presence of downstream components support a revised evolutionary hypothesis. An ancestral EDARADD-related adaptor may have participated in immune or tissue-homeostatic signaling before its recruitment into vertebrate appendage development. Under this model, developmental signaling could have incorporated components of an older immune-signaling network. This is a plausible interpretation of the combined evidence, rather than a demonstrated sequence of evolutionary events. EDARADD’s immediate ancestral gene, its original receptor partners and the function of the lancelet homolog remain unresolved.

We therefore distinguish the origin of an adaptor protein, the assembly of a functional signaling pathway and recruitment of that pathway into appendage development. The relevant developmental innovation could have involved altered receptor–adaptor coupling, spatial and temporal expression, downstream target selection or integration with other signaling pathways. Such changes need not have required newly evolved genes.

We propose that lamprey keratinous toothlets **may have recruited pre-existing EDARADD-related signaling machinery, potentially derived from an older immune-signaling context, together with additional regulatory and developmental inputs**.

The comparative evidence makes this role plausible, although direct functional testing in lamprey is still needed. These structures form part of the lamprey’s feeding apparatus and are compatible with its aggressive predatory lifestyle. We describe them as *keratinous toothlets* rather than *proto-teeth* to distinguish their established feeding function from their unresolved evolutionary relationship to the mineralized teeth of jawed vertebrates [9].

The presence of candidate signaling components in lancelets does not, by itself, predict the formation of appendage-producing placodes. Development requires their coordinated deployment in an appropriate tissue context. In mouse skin, for example, increased EDA or EDAR activity cannot restore hair-follicle development when epithelial Wnt/β-catenin signaling is absent. Whether comparable dependencies operate during lamprey toothlet formation remains to be established [10].

Finally, failure to identify confident EDARADD orthologs in the tunicate and hagfish datasets examined should be described as non-detection, rather than demonstrated absence. The revised interpretation proposes recruitment and modification of older signaling machinery, rather than pathway completion through the first appearance of EDARADD in cyclostomes. Testing this model would require direct interaction assays, including mutation of the candidate TRAF6-binding motifs, developmental expression studies of the lamprey EDARADD-related gene, as well as functional perturbation of the EDAR-EDARADD pathway during lamprey toothlet formation.
