# Supplementary material for: The emergence of the ectodysplasin pathway
Source: Open Life Sci. 2026 Jul 14;21(1):20251345. doi: 10.1515/biol-2025-1345 (PMC13367198; doi:10.1515/biol-2025-1345)
Supplement: Supplementary file 1 — Supplementary Material [file j_biol-2025-1345_suppl_001.docx]

| **Ensembl ID** | **ENSG00000158813** | **ENSG00000135960** | **ENSG00000131080** | **ENSG00000186197** |
| --- | --- | --- | --- | --- |
| **Gene** | **EDA** | **EDAR** | **EDA2R** | **EDARADD** |
| **adipose tissue** | 4 | 0 | 2 | 0.4 |
| **adrenal gland** | 8.3 | 0 | 5.6 | 0.1 |
| **amygdala** | 1.2 | 0 | 0.8 | 0.1 |
| **appendix** | 0.6 | 0.8 | 0.5 | 0.3 |
| **basal ganglia** | 1.1 | 0 | 0.6 | 0.1 |
| **bone marrow** | 0.1 | 0.3 | 0 | 0.4 |
| **breast** | 2.7 | 0.2 | 2.9 | 4.6 |
| **cerebellum** | 0.5 | 0 | 0.1 | 0 |
| **cerebral cortex** | 1 | 0 | 1.8 | 0.1 |
| **cervix** | 4.8 | 0.5 | 5.9 | 0.7 |
| **choroid plexus** | 3.1 | 0 | 3 | 0 |
| **colon** | 2.5 | 1.2 | 2.1 | 0.3 |
| **duodenum** | 0.5 | 1 | 0.3 | 0.4 |
| **endometrium** | 2.9 | 0.5 | 5.5 | 0.3 |
| **epididymis** | 3.1 | 0.2 | 4.1 | 5.1 |
| **esophagus** | 1.5 | 3.5 | 2.3 | 3.2 |
| **fallopian tube** | 3.7 | 0.2 | 4.2 | 1.2 |
| **gallbladder** | 1.9 | 0.9 | 3.2 | 0.8 |
| **heart muscle** | 10 | 0 | 2 | 0.9 |
| **hippocampal formation** | 1.3 | 0 | 1 | 0.1 |
| **hypothalamus** | 1.2 | 0 | 0.6 | 0.1 |
| **kidney** | 3.5 | 0.6 | 1.2 | 1 |
| **liver** | 2 | 0.9 | 0.8 | 0.3 |
| **lung** | 3.1 | 0.2 | 2.5 | 0.6 |
| **lymph node** | 0.8 | 1.5 | 0.5 | 1.2 |
| **midbrain** | 1.3 | 0 | 1.3 | 0.2 |
| **ovary** | 4.6 | 0 | 5.7 | 0.5 |
| **pancreas** | 4.1 | 0.1 | 3 | 3.7 |
| **parathyroid gland** | 9.4 | 0 | 2.9 | 0.1 |
| **pituitary gland** | 1.2 | 0 | 3.3 | 0.1 |
| **placenta** | 1.8 | 0.1 | 1.1 | 4.7 |
| **prostate** | 3.3 | 0.7 | 3.9 | 3.5 |
| **rectum** | 1 | 1.9 | 4.8 | 0.3 |
| **retina** | 0.3 | 0 | 0.6 | 0 |
| **salivary gland** | 2.1 | 0.5 | 2.4 | 1.3 |
| **seminal vesicle** | 2.2 | 0.1 | 5.2 | 5.9 |
| **skeletal muscle** | 1.6 | 0 | 2.4 | 0 |
| **skin** | 4.1 | 1.4 | 1.5 | 5.6 |
| **small intestine** | 1 | 1 | 1.1 | 0.3 |
| **smooth muscle** | 2 | 0 | 5 | 0.1 |
| **spinal cord** | 1.2 | 0 | 1.7 | 0.1 |
| **spleen** | 1.4 | 0.2 | 1.1 | 1.2 |
| **stomach** | 3.7 | 0.8 | 0.8 | 6.1 |
| **testis** | 1.1 | 0.1 | 1.4 | 3.3 |
| **thymus** | 1.2 | 1.1 | 2.5 | 2.2 |
| **thyroid gland** | 5.8 | 0 | 5.5 | 6.3 |
| **tongue** | 2 | 0 | 0.8 | 0 |
| **tonsil** | 0.5 | 1.2 | 0.4 | 2.5 |
| **urinary bladder** | 2.2 | 1.4 | 2.3 | 8.4 |
| **vagina** | 3.8 | 1.4 | 4.1 | 1 |
| **Average** | 2.566 | 0.49 | 2.366 | 1.594 |
| **Rank** | 14671 | 17651 | 14883 | 15794 |
| **%Rank** | 27.2 | 12.5 | 26.2 | 21.7 |
| **Gene** | **EDA** | **EDAR** | **EDA2R** | **EDARADD** |

**Supplementary Table S1.** Human gene expression data for 50 tissue types were downloaded from The Human Protein Atlas project version 24.0 (<https://www.proteinatlas.org/>).

Numbers depicted are normalized transcripts per million (TPM).
