# Supplementary material for: The emergence of the ectodysplasin pathway
Source: Open Life Sci. 2026 Jul 14;21(1):20251345. doi: 10.1515/biol-2025-1345 (PMC13367198; doi:10.1515/biol-2025-1345)
Supplement: Supplementary file 2 — Supplementary Material [file j_biol-2025-1345_suppl_002.docx]

**Supplementary: Sequences and alignments**

| **UniProt_ID (Gene, organism)** | **Sequence (FASTA format)** |
| --- | --- |
| [Q92838](https://www.uniprot.org/uniprotkb/Q92838/entry)  EDA, Homo sapiens | >sp\|Q92838\|EDA_HUMAN Ectodysplasin-A OS=Homo sapiens OX=9606 GN=EDA PE=1 SV=2  MGYPEVERRELLPAAAPRERGSQGCGCGGAPARAGEGNSCLLFLGFFGLSLALHLLTLCC YLELRSELRRERGAESRLGGSGTPGTSGTLSSLGGLDPDSPITSHLGQPSPKQQPLEPGE  AALHSDSQDGHQMALLNFFFPDEKPYSEEESRRVRRNKRSKSNEGADGPVKNKKKGKKAG PPGPNGPPGPPGPPGPQGPPGIPGIPGIPGTTVMGPPGPPGPPGPQGPPGLQGPSGAADK  AGTRENQPAVVHLQGQGSAIQVKNDLSGGVLNDWSRITMNPKVFKLHPRSGELEVLVDGT YFIYSQVEVYYINFTDFASYEVVVDEKPFLQCTRSIETGKTNYNTCYTAGVCLLKARQKI  AVKMVHADISINMSKHTTFFGAIRLGEAPAS |
| [Q9UNE0](https://www.uniprot.org/uniprotkb/Q9UNE0/entry)  EDAR, Homo sapiens | >sp\|Q9UNE0\|EDAR_HUMAN Tumor necrosis factor receptor superfamily member EDAR OS=Homo sapiens OX=9606 GN=EDAR PE=1 SV=1  MAHVGDCTQTPWLPVLVVSLMCSARAEYSNCGENEYYNQTTGLCQECPPCGPGEEPYLSC GYGTKDEDYGCVPCPAEKFSKGGYQICRRHKDCEGFFRATVLTPGDMENDAECGPCLPGY  YMLENRPRNIYGMVCYSCLLAPPNTKECVGATSGASANFPGTSGSSTLSPFQHAHKELSG QGHLATALIIAMSTIFIMAIAIVLIIMFYILKTKPSAPACCTSHPGKSVEAQVSKDEEKK  EAPDNVVMFSEKDEFEKLTATPAKPTKSENDASSENEQLLSRSVDSDEEPAPDKQGSPEL CLLSLVHLAREKSATSNKSAGIQSRRKKILDVYANVCGVVEGLSPTELPFDCLEKTSRML  SSTYNSEKAVVKTWRHLAESFGLKRDEIGGMTDGMQLFDRISTAGYSIPELLTKLVQIER LDAVESLCADILEWAGVVPPASQPHAAS |
| [Q8WWZ3](https://www.uniprot.org/uniprotkb/Q8WWZ3/entry) EDARADD, Homo sapiens | >sp\|Q8WWZ3\|EDAD_HUMAN Ectodysplasin-A receptor-associated adapter protein OS=Homo sapiens OX=9606 GN=EDARADD PE=1 SV=3  MGLRTTKQMGRGTKAPGHQEDHMVKEPVEDTDPSTLSFNMSDKYPIQDTELPKAEECDTI TLNCPRNSDMKNQGEENGFPDSTGDPLPEISKDNSCKENCTCSSCLLRAPTISDLLNDQD  LLDVIRIKLDPCHPTVKNWRNFASKWGMSYDELCFLEQRPQSPTLEFLLRNSQRTVGQLM ELCRLYHRADVEKVLRRWVDEEWPKRERGDPSRHF |
| [Q9HAV5](https://www.uniprot.org/uniprotkb/Q9HAV5/entry) EDA2R (XEDAR), Homo sapiens | >sp\|Q9HAV5\|TNR27_HUMAN Tumor necrosis factor receptor superfamily member 27 OS=Homo sapiens OX=9606 GN=EDA2R PE=1 SV=2  MDCQENEYWDQWGRCVTCQRCGPGQELSKDCGYGEGGDAYCTACPPRRYKSSWGHHRCQS CITCAVINRVQKVNCTATSNAVCGDCLPRFYRKTRIGGLQDQECIPCTKQTPTSEVQCAF  QLSLVEADTPTVPPQEATLVALVSSLLVVFTLAFLGLFFLYCKQFFNRHCQRGGLLQFEA DKTAKEESLFPVPPSKETSAESQVSENIFQTQPLNPILEDDCSSTSGFPTQESFTMASCT  SESHSHWVHSPIECTELDLQKFSSSASYTGAETLGGNTVESTGDRLELNVPFEVPSP |
| [Q9NS68](https://www.uniprot.org/uniprotkb/Q9NS68/entry) TNFRSF19 (TROY) Homo sapiens | >sp\|Q9NS68\|TNR19_HUMAN Tumor necrosis factor receptor superfamily member 19 OS=Homo sapiens OX=9606 GN=TNFRSF19 PE=1 SV=1  MALKVLLEQEKTFFTLLVLLGYLSCKVTCESGDCRQQEFRDRSGNCVPCNQCGPGMELSK ECGFGYGEDAQCVTCRLHRFKEDWGFQKCKPCLDCAVVNRFQKANCSATSDAICGDCLPG  FYRKTKLVGFQDMECVPCGDPPPPYEPHCASKVNLVKIASTASSPRDTALAAVICSALAT VLLALLILCVIYCKRQFMEKKPSWSLRSQDIQYNGSELSCFDRPQLHEYAHRACCQCRRD  SVQTCGPVRLLPSMCCEEACSPNPATLGCGVHSAASLQARNAGPAGEMVPTFFGSLTQSI CGEFSDAWPLMQNPMGGDNISFCDSYPELTGEDIHSLNPELESSTSLDSNSSQDLVGGAV  PVQSHSENFTAATDLSRYNNTLVESASTQDALTMRSQLDQESGAVIHPATQTSLQVRQRL GSL |
| [P09958](https://www.uniprot.org/uniprotkb/P09958/entry) FURIN, Homo sapiens | >sp\|P09958\|FURIN_HUMAN Furin OS=Homo sapiens OX=9606 GN=FURIN PE=1 SV=2  MELRPWLLWVVAATGTLVLLAADAQGQKVFTNTWAVRIPGGPAVANSVARKHGFLNLGQI FGDYYHFWHRGVTKRSLSPHRPRHSRLQREPQVQWLEQQVAKRRTKRDVYQEPTDPKFPQ  QWYLSGVTQRDLNVKAAWAQGYTGHGIVVSILDDGIEKNHPDLAGNYDPGASFDVNDQDP DPQPRYTQMNDNRHGTRCAGEVAAVANNGVCGVGVAYNARIGGVRMLDGEVTDAVEARSL  GLNPNHIHIYSASWGPEDDGKTVDGPARLAEEAFFRGVSQGRGGLGSIFVWASGNGGREH DSCNCDGYTNSIYTLSISSATQFGNVPWYSEACSSTLATTYSSGNQNEKQIVTTDLRQKC  TESHTGTSASAPLAAGIIALTLEANKNLTWRDMQHLVVQTSKPAHLNANDWATNGVGRKV SHSYGYGLLDAGAMVALAQNWTTVAPQRKCIIDILTEPKDIGKRLEVRKTVTACLGEPNH  ITRLEHAQARLTLSYNRRGDLAIHLVSPMGTRSTLLAARPHDYSADGFNDWAFMTTHSWD EDPSGEWVLEIENTSEANNYGTLTKFTLVLYGTAPEGLPVPPESSGCKTLTSSQACVVCE  EGFSLHQKSCVQHCPPGFAPQVLDTHYSTENDVETIRASVCAPCHASCATCQGPALTDCL SCPSHASLDPVEQTCSRQSQSSRESPPQQQPPRLPPEVEAGQRLRAGLLPSHLPEVVAGL  SCAFIVLVFVTVFLVLQLRSGFSFRGVKVYTMDRGLISYKGLPPEAWQEECPSDSEEDEG RGERTAFIKDQSAL |
| [P01106](https://www.uniprot.org/uniprotkb/P01106/entry) MYC, Homo sapiens | >sp\|P01106\|MYC_HUMAN Myc proto-oncogene protein OS=Homo sapiens OX=9606 GN=MYC PE=1 SV=2  MDFFRVVENQQPPATMPLNVSFTNRNYDLDYDSVQPYFYCDEEENFYQQQQQSELQPPAP SEDIWKKFELLPTPPLSPSRRSGLCSPSYVAVTPFSLRGDNDGGGGSFSTADQLEMVTEL  LGGDMVNQSFICDPDDETFIKNIIIQDCMWSGFSAAAKLVSEKLASYQAARKDSGSPNPA RGHSVCSTSSLYLQDLSAAASECIDPSVVFPYPLNDSSSPKSCASQDSSAFSPSSDSLLS  STESSPQGSPEPLVLHEETPPTTSSDSEEEQEDEEEIDVVSVEKRQAPGKRSESGSPSAG GHSKPPHSPLVLKRCHVSTHQHNYAAPPSTRKDYPAAKRVKLDSVRVLRQISNNRKCTSP  RSSDTEENVKRRTHNVLERQRRNELKRSFFALRDQIPELENNEKAPKVVILKKATAYILS VQAEEQKLISEEDLLRKRREQLKHKLEQLRNSCA |
| [P04198](https://www.uniprot.org/uniprotkb/P04198/entry) MYCN, Homo sapiens | >sp\|P04198\|MYCN_HUMAN N-myc proto-oncogene protein OS=Homo sapiens OX=9606 GN=MYCN PE=1 SV=2  MPSCSTSTMPGMICKNPDLEFDSLQPCFYPDEDDFYFGGPDSTPPGEDIWKKFELLPTPP LSPSRGFAEHSSEPPSWVTEMLLENELWGSPAEEDAFGLGGLGGLTPNPVILQDCMWSGF  SAREKLERAVSEKLQHGRGPPTAGSTAQSPGAGAASPAGRGHGGAAGAGRAGAALPAELA HPAAECVDPAVVFPFPVNKREPAPVPAAPASAPAAGPAVASGAGIAAPAGAPGVAPPRPG  GRQTSGGDHKALSTSGEDTLSDSDDEDDEEEDEEEEIDVVTVEKRRSSSNTKAVTTFTIT VRPKNAALGPGRAQSSELILKRCLPIHQQHNYAAPSPYVESEDAPPQKKIKSEASPRPLK  SVIPPKAKSLSPRNSDSEDSERRRNHNILERQRRNDLRSSFLTLRDHVPELVKNEKAAKV VILKKATEYVHSLQAEEHQLLLEKEKLQARQQQLLKKIEHARTC |
| [P12524](https://www.uniprot.org/uniprotkb/P12524/entry) LMYC, Homo sapiens | >sp\|P12524\|MYCL_HUMAN Protein L-Myc OS=Homo sapiens OX=9606 GN=MYCL PE=1 SV=2  MDYDSYQHYFYDYDCGEDFYRSTAPSEDIWKKFELVPSPPTSPPWGLGPGAGDPAPGIGP PEPWPGGCTGDEAESRGHSKGWGRNYASIIRRDCMWSGFSARERLERAVSDRLAPGAPRG  NPPKASAAPDCTPSLEAGNPAPAAPCPLGEPKTQACSGSESPSDSENEEIDVVTVEKRQS LGIRKPVTITVRADPLDPCMKHFHISIHQQQHNYAARFPPESCSQEEASERGPQEEVLER  DAAGEKEDEEDEEIVSPPPVESEAAQSCHPKPVSSDTEDVTKRKNHNFLERKRRNDLRSR FLALRDQVPTLASCSKAPKVVILSKALEYLQALVGAEKRMATEKRQLRCRQQQLQKRIAY LTGY |
| [Q9Y275](https://www.uniprot.org/uniprotkb/Q9Y275/entry) BAFF, Homo sapiens | >sp\|Q9Y275\|TN13B_HUMAN Tumor necrosis factor ligand superfamily member 13B OS=Homo sapiens OX=9606 GN=TNFSF13B PE=1 SV=1  MDDSTEREQSRLTSCLKKREEMKLKECVSILPRKESPSVRSSKDGKLLAATLLLALLSCC LTVVSFYQVAALQGDLASLRAELQGHHAEKLPAGAGAPKAGLEEAPAVTAGLKIFEPPAP  GEGNSSQNSRNKRAVQGPEETVTQDCLQLIADSETPTIQKGSYTFVPWLLSFKRGSALEE KENKILVKETGYFFIYGQVLYTDKTYAMGHLIQRKKVHVFGDELSLVTLFRCIQNMPETL  PNNSCYSAGIAKLEEGDELQLAIPRENAQISLDGDVTFFGALKLL |
| [O75888](https://www.uniprot.org/uniprotkb/O75888/entry) TNFSF13 (APRIL) Homo sapiens | >sp\|O75888\|TNF13_HUMAN Tumor necrosis factor ligand superfamily member 13 OS=Homo sapiens OX=9606 GN=TNFSF13 PE=1 SV=1  MPASSPFLLAPKGPPGNMGGPVREPALSVALWLSWGAALGAVACAMALLTQQTELQSLRR EVSRLQGTGGPSQNGEGYPWQSLPEQSSDALEAWENGERSRKRRAVLTQKQKKQHSVLHL  VPINATSKDDSDVTEVMWQPALRRGRGLQAQGYGVRIQDAGVYLLYSQVLFQDVTFTMGQ VVSREGQGRQETLFRCIRSMPSHPDRAYNSCYSAGVFHLHQGDILSVIIPRARAKLNLSP  HGTFLGFVKL |
| [O43508](https://www.uniprot.org/uniprotkb/O43508/entry) TNFS12 (TWEAK) Homo sapiens | >sp\|O43508\|TNF12_HUMAN Tumor necrosis factor ligand superfamily member 12 OS=Homo sapiens OX=9606 GN=TNFSF12 PE=1 SV=1  MAARRSQRRRGRRGEPGTALLVPLALGLGLALACLGLLLAVVSLGSRASLSAQEPAQEEL VAEEDQDPSELNPQTEESQDPAPFLNRLVRPRRSAPKGRKTRARRAIAAHYEVHPRPGQD  GAQAGVDGTVSGWEEARINSSSPLRYNRQIGEFIVTRAGLYYLYCQVHFDEGKAVYLKLD LLVDGVLALRCLEEFSATAASSLGPQLRLCQVSGLLALRPGSSLRIRTLPWAHLKAAPFL  TYFGLFQVH |
| **Supplementary Table 1.** List of human sequences of interest. Multiple isoforms are produced by alternative splicing, but here, only the canonical sequence is reported. The UniProt_ID links to the corresponding entry in [UniProt](https://www.uniprot.org), which provides detailed sequence, family, domain, region, and functional information. | |

| **Protein_ID (Gene, organism)** | **Sequence (FASTA format)** |
| --- | --- |
| [CAB3240801.1](https://www.ncbi.nlm.nih.gov/protein/CAB3240801.1/)  EDA Phallusia mammillata | >CAB3240801.1 ectodysplasin-A [Phallusia mammillata]  MGTNNNTMNVSDVSDSEALHSCYTCEHSGTKKSYCVKSNRQKNRQRGRLCAKWPCFCLIA LSIICAVMAIFSVFIYIQLSQEINQLKTNFKVLQKRCGTNTNLQVVEGSLAKLVGETKRN ENSVSFGATPKVGEKVTRPRRHDGHPHDGFHTHGRDGRDGRDGANGAMGPPGPPGPPGLP GEGVIGPLGPVGPPGPQGLPGLPGIAGPPGQSVSMSLIHLQGDGSSNVPSNSHGTLNHWS RTTWSSGDDFRYFQHNGTVKVLKSGIYYIYSQLLTNESPTTSLSHEVKINHRTFLKCKQA VKRSTCFTAGVRKLRAHDTVHIVATYPKTKVDMHEDVSFFGLVQWNQQGHDEL |
| [**CAH1267392.1**](https://www.ncbi.nlm.nih.gov/protein/CAH1267392.1) EDAR Branchiostoma lanceolatum | >CAH1267392.1 EDAR [Branchiostoma lanceolatum]  MQLTPVTGANWIVPLSLLYILEVGARQVPCGSEEFLQAGSCRPCPQCPDGEQLSADCGHG LGAGAVCVPCPPDHYSNADTRHQCWPCTDCSLENKVQEWSCTPTHNADCGRCLEGYFQFA GLHCHLCSIEPTNPSCKDWLSQQTTTQQSTAIPWEASSSTHIKQDIVTESYGQHDIDLPG KITGIAFVVIIVVVVLIVTAVYIAKNWEHILRCCAEPHENSTVQEPEKQDLGTMERNTSA SQHGGVINHSHLHLADQSSANLGSSNTEEESGLTSDGAAASPSDVGVRCSAQKSQNGQAA NYLADLKKKALEATADKKVEDLKQDPRDVLARDLFSEPPAAGVHKNWRHFADYFNIEKTY IDNWKDRNDPMMHVLEHLEKSTQVTVPEIIEAIYSIERVQVLERFCHSLLKEYN |
| [XP_030833294.1](https://www.ncbi.nlm.nih.gov/protein/XP_030833294.1) EDA2R Strongylocentrotus purpuratus | >XP_030833294.1 tumor necrosis factor receptor superfamily member 27 isoform X2 [Strongylocentrotus purpuratus]  MTIAMEFCDHKLEISGWNLKVRLLLQVSLKLLCLQLASGSVQALCNGELEIDPQAPGCLD DCEGRRFFLGRDGMCKMCVQCEPGFEHDKFCGDGENGDAVCIPCPEGHYSYNRYECLACR VCEKTVLTRECSATTDRQCGECLQGFYRDPQNPDLCNQRCEWLSNPKPTLHAWLGWQISK NQLNLPSYPRHLHFLCFQLKGLEVCL |
| [CAB3264104.1](https://www.ncbi.nlm.nih.gov/protein/CAB3264104.1) MyD88 Phallusia mammillata | >CAB3264104.1 myeloid differentiation primary response protein MyD88 [Phallusia mammillata]  MAEEKKLQPEDLDEMASIQMQRKEMESFSSGYEKPPIDTPVECDSTNTDVAGQSSLGRRL AGVQESGFDSGKSATSMPTLDASTHLHSPPPSYYNQPEGSWSSDQSTNLEKKDEVNLVAT YGMGRPSDDYMSMPLKKLRYETRRQLGLHLDPDRPTVPNWKSVADYLGFSNLQIQNFEQD RYKTQAVLNECEVRQPNLTVGDLCKILQELGRLDVITDLQECFERDYKEPPLNSLVSARF DQSPAPSYRTVEPLQECAVSSTFYDHQQKTDIPPHLPEERYDAFVLYNIADKDFILKEFL PKVEVDAQIKLFLPERDLTAGVQSYHAKLNTMIGRCSKLIIILSNEFLTSPESQWALNMG VSLDPASRMQKIIPVIYKTITEPNILGGYNACDRTRVLEDSWFWNNVIKSLKRGRV |
| [XP_048588342.1](https://www.ncbi.nlm.nih.gov/protein/XP_048588342.1) EDA isoform X3 Nematostella vectensis | >XP_048588342.1 ectodysplasin-A isoform X3 [Nematostella vectensis]  MESESDGYDNGEKLPEEKVYYQDSRSCSCGGGGRAAITNNLPLISLVFNVVFVVLFVLVF LRLENVKTRLSVLESREALAVNGAEVRASSRQQVGLGPIASNTARSNTNTNRTASMWETS NSTTNPTESVAKSTTLASKNHAANSVWKRSTSTAKPTTPSRVLPKKLLRKWMRKDINRLR TELCTCPRGPRGRRGKTGLPGPPGSPGSNGHAGMPGQRGLDGPRGPAGLKGDPGPVGPKG DKGDTGSQGPPGKRGPPGEKGDQGIPGVKGDVGPKGEKGDSGGPEPPPRPSAHLTGYHKS QQNAPRTGILRHWEDSIGYAHSFGGMHYRNGELIIPTSGRYYVYSQLYFQAEDDKPHMIH MHLTANNVTSVIMRSVTSRCRARKAKAHLFSSYQGGVFLLAAGNKLSVGVSEGQSDTVAM GESASFFGAFMI |
| [XP_001638170.1](https://www.ncbi.nlm.nih.gov/protein/XP_001638170.1?report=genpept) EDAR like Nematostella vectensis | >XP_001638170.1 predicted protein [Nematostella vectensis] MPTTERRNSERILLCVTVSVVLLFVVLTTVFIALQGRQAIAQSARMDELEGSIRRLSRRV DVLEKELLTARDHPSRDEGGRGDANTETRRKDISTDPTARKRRQFSSYCPCVQGPQGPNG TRGLQGEKGPQGDQGPKGEPGTPGAKGESGAQGPPGRVDAESIHLVGDNQAVIQPSASQN IKAWRLKHKQGSIQYRPLTGMIEIAAPAISQSFVNWRPAHKQGSIRYHRLTGSIIIHHPG RLRVGAWRLDHRIGSIQYRRRTHEIQIAIAGYYFVYSQVMYYDDKAYRVSYGVFVNRYQV LGGSLHSPEVKYNSMFSGGVLKLQAGDLLSVRLTRRAPTKLKMNSTITFALYTTQELTDI KTRLYELEGSQTAAKEPGNKTEESKHAKRSLNQNLSSRVRSLPGGVAIHVVGDNTLKTMP QPHRHVDYRQVMGWRVMRSTGSIHFDANWGYITVYTPGYYFIYSQINYASGGLTSHVTLV NSKTVLRSITSSSKRNKPQTNYQGGVFLLSDGDRISIRIPFVHKRFNMNAAASYFGAFLV AAV |
| [XP_048588379.1](https://www.ncbi.nlm.nih.gov/protein/XP_048588379.1) EDA Nematostella vectensis | >XP_048588379.1 ectodysplasin-A [Nematostella vectensis]  MTNMPEVNSFTFFVTGPQGPNGTRGLQGEKGPQGDQGPKGEPGTPGAKGESGAQGPPGRV DAESIHLVGDNQAVIQPWRLRVGAWRLDHRIGSIQYRRRTHEIQIAIAGYYFVYSQVMYY DDKAYRVSYGVFVNRYQVLGGSLHSPEVKYNSMFSGGVLKLQAGDLLSVRLTRRAPTKLK MNSSMSYFGAFLVHGNPAKAIGQPS |
| [XP_001632565.1](https://www.ncbi.nlm.nih.gov/protein/XP_001632565.1?report=genpept) EDA2R (XEDAR) like Nematostella vectensis | >XP_001632565.1 predicted protein [Nematostella vectensis]  MRCKYNQIEWVKHGKIVACQACPQCTPGEESSLMCGTRIESGTLSVCRPCKEGTYSDRFD AIPCKPCTRCSTGRIILRNCTSTENAKCGKCMSGYYELPVVADCRKCVQCCHDGKDKETP GCAVVGLCMERHEGCPETTPRTPNVPSTSPEPTTRRSTSTESTSSQSTPSPASSVTQLLD VTRSKTGHDTSSVTTLPVSQTPTSDPTTQLQSGDSNTYKYVAIGSTMGLVIVVLIGFFCK MKGNWNQNIPSDVLSVQLPFLQRDTEVELNKAVDWLNGNLGSGAYENAQDACTELDKEKR GTYQYVAVGVRFLKTKTELDRHRCVTRGGARGVNHPLDRQNKD |
| **Supplementary Table 2.** List of all non-human sequences of interest. The Protein_ID links to the corresponding [NCBI GenBank](https://www.ncbi.nlm.nih.gov) entry. | |

| **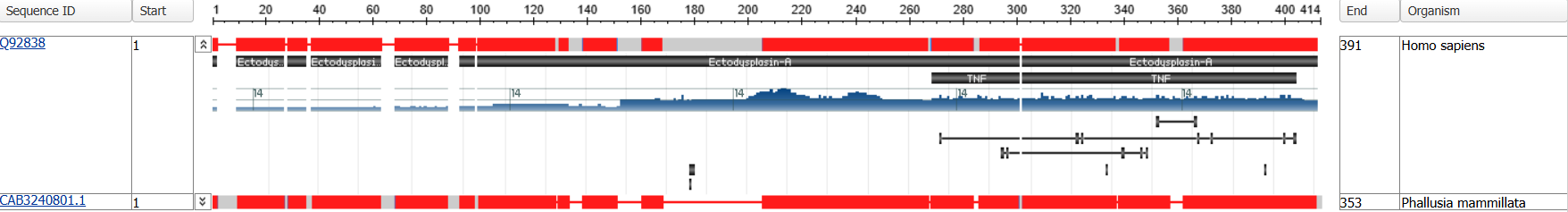** |
| --- |
| **Supplementary Figure 1.** Alignment between **EDA** from Homo sapiens (including domain annotation) and Phallusia mammillata. |

| **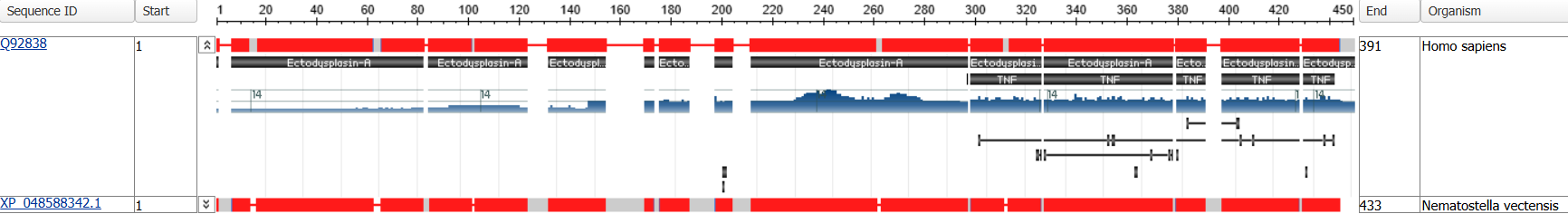** |
| --- |
| **Supplementary Figure 2.** Alignment between **EDA** from Homo sapiens (including domain annotation) and EDA isoform X3 from Nematostella vectensis. |

| **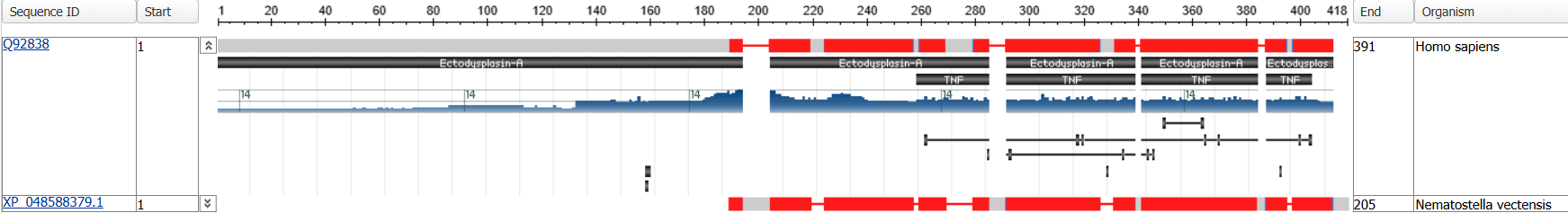** |
| --- |
| **Supplementary Figure 3.** Alignment between **EDA** from Homo sapiens (including domain annotation) and an EDA-like protein from Nematostella vectensis. |

| 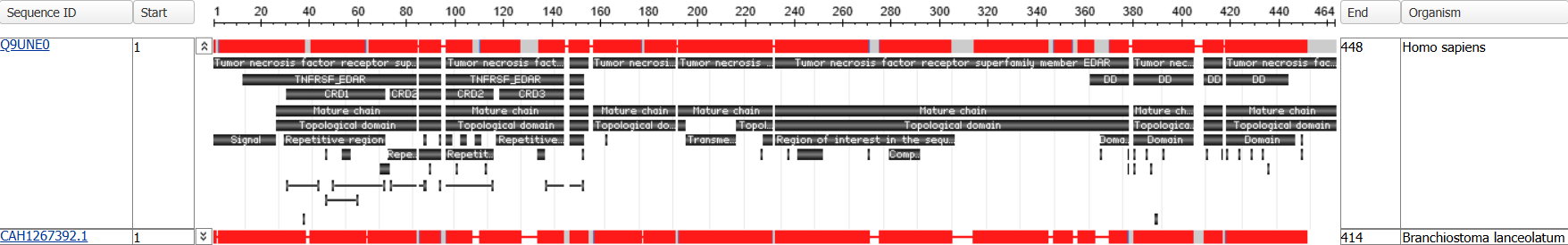 |
| --- |
| **Supplementary Figure 4.** Alignment between **EDAR** from Homo sapiens (including domain annotation) and Branchiostoma lanceolatum. |

| **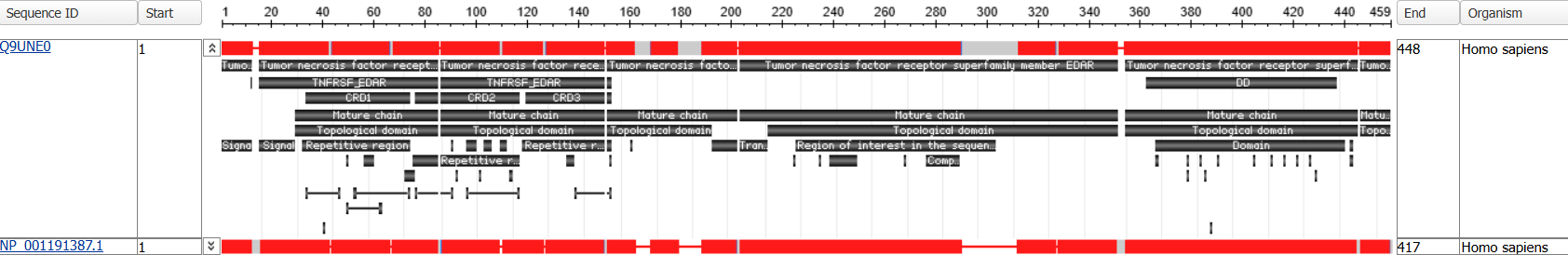** |
| --- |
| **Supplementary Figure 5**. Alignment between human **EDAR** (including domain annotation) and TNFRSF19 (TROY). |

| **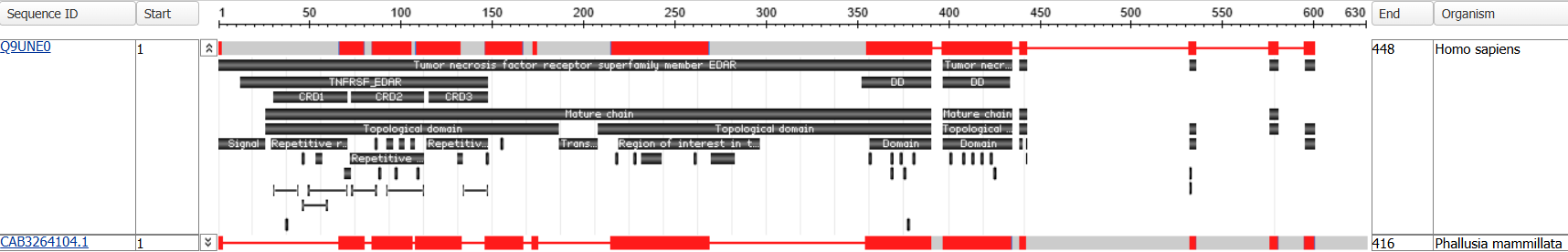** 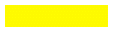 |
| --- |
| **Supplementary Figure 6.** Alignment between **EDAR** from Homo sapiens (including domain annotation, the death domain is highlighted in yellow) and the EDAR homologue MyD88 from Phallusia mammillata. |

| **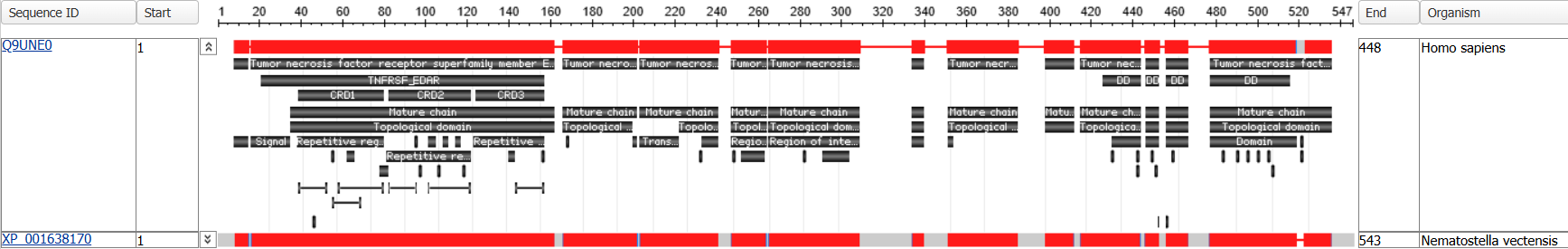** |
| --- |
| **Supplementary Figure 7.** Alignment between **EDAR** from Homo sapiens (including domain annotation) and an EDAR-like protein from Nematostella vectensis. |

| **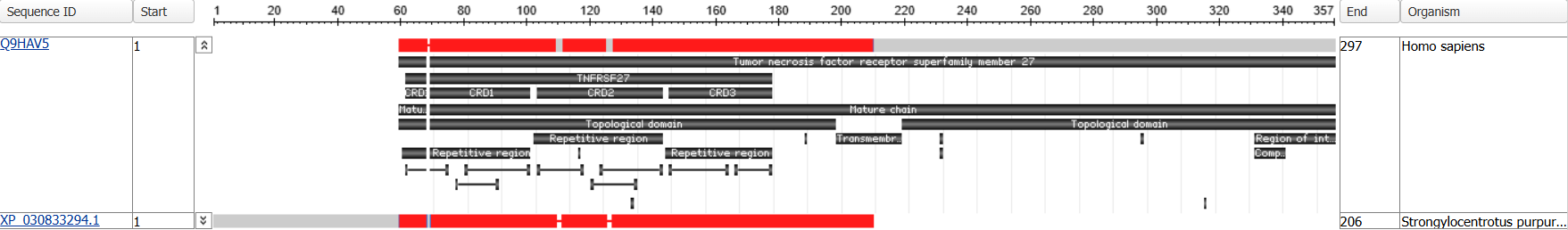** |
| --- |
| **Supplementary Figure 8**. Alignment between **EDA2R** (XEDAR) from Homo sapiens (including domain annotation) and Strongylocentrotus purpuratus. |

| **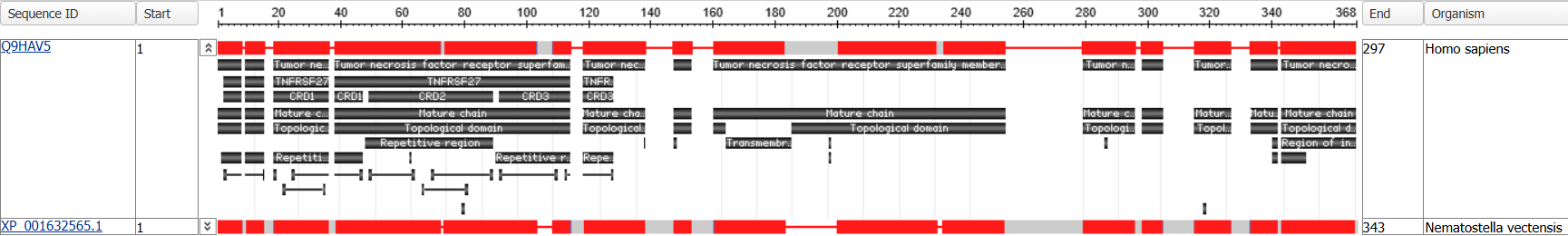** |
| --- |
| **Supplementary Figure 9.** Alignment between **EDA2R (XEDAR)** from Homo sapiens and an EDA2R like protein from Nematostella vectensis. |
